# Transient fusion and selective secretion of vesicle proteins in *Phytophthora nicotianae* zoospores

**DOI:** 10.7717/peerj.221

**Published:** 2013-12-03

**Authors:** Weiwei Zhang, Leila M. Blackman, Adrienne R. Hardham

**Affiliations:** Plant Science Division, Research School of Biology, College of Medicine, Biology and Environment, The Australian National University, Canberra, ACT, Australia

**Keywords:** Dynamin, Kiss-and-run, Sub-vesicle compartmentalization, Regulated secretion, Transient fusion

## Abstract

Secretion of pathogen proteins is crucial for the establishment of disease in animals and plants. Typically, early interactions between host and pathogen trigger regulated secretion of pathogenicity factors that function in pathogen adhesion and host penetration. During the onset of plant infection by spores of the Oomycete, *Phytophthora nicotianae*, proteins are secreted from three types of cortical vesicles. Following induction of spore encystment, two vesicle types undergo full fusion, releasing their entire contents onto the cell surface. However, the third vesicle type, so-called large peripheral vesicles, selectively secretes a small Sushi domain-containing protein, PnCcp, while retaining a large glycoprotein, PnLpv, before moving away from the plasma membrane. Selective secretion of PnCcp is associated with its compartmentalization within the vesicle periphery. Pharmacological inhibition of dynamin function, purportedly in vesicle fission, by dynasore treatment provides evidence that selective secretion of PnCcp requires transient fusion of the large peripheral vesicles. This is the first report of selective protein secretion via transient fusion outside mammalian cells. Selective secretion is likely to be an important aspect of plant infection by this destructive pathogen.

## Introduction

Secretion is a fundamental process essential for many aspects of development including cell adhesion, migration, communication and differentiation. Secretion also plays a vital role in the establishment of disease in animals and plants. During host-pathogen interactions, prokaryotic and eukaryotic pathogens release a wide range of pathogenicity factors that are required for infection. In malaria parasites such as *Plasmodium* and *Toxoplasma* species, for example, successful invasion of host cells is dependent upon the regulated secretion of proteins located in three categories of apical vesicles (Carruthers & Sibley, 1997). Fungal pathogens of plants also secrete a wide range of effector proteins that facilitate infection by aiding adhesion and penetration or by suppressing the plant defence response (Gan et al., 2010; Koeck, Hardham & Dodds, 2011). In addition, secretion plays a vital role in host immunity because potential hosts release a range of antimicrobial compounds as part of their defence against pathogen attack.

Protein secretion is a key feature in the establishment of disease in animals and plants by Oomycete pathogens. Oomycetes are morphologically similar to filamentous fungi but are phylogenetically related to organisms, including the malarial parasites, in the Kingdom Protista (Simpson, Inagaki & Roger, 2006). Oomycete species are responsible for extensive losses in salmon and crayfish populations and for debilitating, sometimes lethal, infections in mammals, including humans (Robertson et al., 2009; Cerenius, Andersson & Söderhäll, 2009; Mendoza, 2009). In addition, over 80 Oomycetes in the *Phytophthora* genus are responsible for highly destructive plant diseases, including many of agricultural importance and some that threaten natural ecosystems on a vast scale (Lamour & Kamoun, 2009; Lamour, 2013). *Phytophthora* disease development is initiated by wall-less, motile spores, termed zoospores. Zoospores swim chemotactically to the surface of potential hosts where they encyst, a process involving secretion of adhesives, mucilages and cell wall material. The cysts subsequently germinate and secrete enzymes that enable host penetration.

During *Phytophthora* zoospore encystment, proteins are secreted from three different types of vesicles that lie next to the plasma membrane within the zoospore cortical cytoplasm (Hardham & Hyde, 1997). The regulated secretion of the contents of small, so-called ventral and dorsal vesicles delivers an adhesin and a putative protective coating, respectively, onto the surface of the cysts (Robold & Hardham, 2005). Secretion of a 10 kDa putative adhesive, a Sushi domain-containing protein named PnCcp, from the third category of cortical vesicle, the so-called large peripheral vesicles, has also been observed (Škalamera & Hardham, 2006). Secretion of PnCcp was unexpected because in previous studies there had been no evidence for the secretion of a high molecular weight glycoprotein, PcLpv, from the large peripheral vesicles nor for large peripheral vesicle exocytosis during zoospore encystment (Gubler & Hardham, 1990). Instead, after encystment, the large peripheral vesicles become randomly distributed throughout the cyst cytoplasm. How could one protein be secreted, another retained and integrity of the large peripheral vesicles maintained during zoospore encystment?

These observations could be explained by the operation of an unusual mode of secretion termed transient fusion or kiss-and-run secretion. In the classical mode of secretion, proteins and glycoproteins destined for the extracellular environment are packaged into vesicles which fuse with and fully collapse into the plasma membrane, thus releasing their entire contents outside the cell. However, there is evidence that in some cases vesicles fuse with the plasma membrane only transiently before the fusion pore closes and the vesicles pinch off and move away (Harata, Aravanis & Tsien, 2006). During this transient fusion, vesicle contents are only partially released. This non-classical mode of secretion is thought to be employed as a mechanism to regulate secretory output and/or rapidly recycle secretory vesicles. It has been documented in mammalian neuronal (He et al., 2006), endothelial (Babich et al., 2008), neuroendocrine and endocrine cells (Taraska et al., 2003; van Kempen et al., 2011) but the extent to which transient fusion occurs outside these mammalian cells is not known. It has been postulated to be present in plant protoplasts but the evidence is limited (Weise et al., 2000; Bandmann, Kreft & Homann, 2011). Does the process of transient fusion explain the apparent differential secretion of proteins from *Phytophthora* large peripheral vesicles or do alternative scenarios fit the data better? In either case, can this novel system reveal new information on the mechanisms that contribute to the regulation of selective protein secretion?

These questions have been addressed in the research reported in this paper. Our results reveal temporal differences in the packaging and spatial differences in the localization of PnCcp and PnLpv proteins in the large peripheral vesicles. Experimental studies indicate that dynamin plays an important role in selective secretion in this system, possibly by regulating fusion pore dynamics. Together, our results indicate that the selective secretion of large peripheral vesicle proteins in *Phytophthora* zoospores utilizes a kiss-and-run, transient fusion mechanism similar to that described in mammalian cells, and they provide new information on factors that may contribute to the mechanics of selective secretion.

## Materials and Methods

### *P. nicotianae* isolate and culture conditions

*P. nicotianae* (Breda de Haan) [isolate H1111 (Gabor et al., 1993); ATCC isolate MYA-141] was grown in V8 broth (Robold & Hardham, 1998). Vegetative and sporulating hyphae, zoospores and 3 h germinated cysts were cultured and harvested as previously described (Gan et al., 2009).

### Expression of recombinant PnCcp and production of polyclonal antibodies

PnCcp was amplified using Ex Taq DNA Polymerase (TaKaRa Bio Inc., Shiga, Japan) from *P. nicotianae* cDNA with primers (TACTGGATCCGCCAATCTTCGTGGAAG and CGTAGAATTCGTTAGCCGGAGTAAAGTG) that removed the signal peptide and placed into the expression vector pGIL (kindly provided by Drs Mark Hinds and Gilles Rautureau). The protein with an N-terminal hexa-histidine-Maltose-Binding Protein (His_6_-MBP) tag was expressed in the *Escherichia coli* host strain AD494(DE3) and purified as previously described (Rafiqi et al., 2010). The His_6_-MBP tag was cleaved with His_6_-tobacco etch virus protease (Tropea et al., 2007). A rabbit was inoculated five times with 0.5 mg of purified PnCcp protein and the resulting polyclonal antiserum was designated as anti-PnCcp^wp^.

### Immunoblot analysis

Purified PnCcp fusion protein was separated by SDS-PAGE and transferred onto Hybond C-extra membranes (GE Healthcare) according to manufacturer’s instructions. Binding of PnCcp^wp^ antiserum and an antibody previously raised against a C-terminal peptide from PnCcp (Škalamera & Hardham, 2006) (designated PnCcp^Cpep^) was detected using goat anti-rabbit IgG (GAR) conjugated to alkaline phosphatase (Merck Millipore). Pre-immune sera and secondary antibodies only were used as controls. Immunoblots of proteins from *P. nicotianae* sporulating hyphae were immunolabelled with monoclonal Lpv-1 antibody followed by goat anti-mouse IgG (GAM) conjugated to alkaline phosphatase.

### Preparation of material for immunolabelling

For surface labelling of *P. nicotianae* zoospores and cysts, spores were fixed in 4% formaldehyde plus 0.2% glutaraldehyde in 50 mM PIPES buffer (Hardham, 1985). For intracellular and surface labelling, zoospores were fixed in 4% formaldehyde in 50 mM PIPES buffer. To detect intracellular cyst proteins, cyst walls were digested with 1 mg/mL cellulase (Sigma-Aldrich) and 20 mg/mL driselase (Sigma-Aldrich) in phosphate buffered saline (PBS, 16 mM Na_2_HPO_4_, 4 mM NaH_2_PO_4_, 150 mM NaCl, pH 7) for 45 min. For immunofluorescence labelling of hyphae, samples were fixed and sectioned as previously described (Dearnaley, Maleszka & Hardham, 1996). For labelling of cysts *in planta*, 2 d lupin (*Lupinus angustifolius* cultivar Gungurru) seedlings were inoculated with a suspension containing 10,000 *P. nicotianae* zoospores/mL for 10 min and root segments were fixed, embedded and cryosectioned as for hyphae.

Labelling of large peripheral vesicles was analyzed in mycelial blocks taken every 5 mm from the center of a colony grown for 5 d on V8 agar. Blocks were fixed in 4% formaldehyde as described above and cryosections double-labelled with PnCcp^Cpep^ and Lpv-1 antibodies. For quantitative analysis, the percentage of vesicles labelled by Lpv-1 only was determined from counts in 100 hyphal fragments in sections from each of three samples from each location.

For ultrastructural studies, *P. nicotianae* sporulating hyphae were cryofixed and freeze-substituted following two protocols. Tufts of hyphae were plunged into liquid propane and freeze-substituted in acetone containing 0.2% uranyl acetate and 1% glutaraldehyde (McDonald & Webb, 2011) overnight at −85°C, washed with acetone at −20°C and slowly infiltrated in Lowicryl K4M before embedding and polymerization under UV light at −20°C. Tufts of hyphae were plunged into liquid ethane and freeze-substituted in acetone containing 0.5% uranyl acetate and 0.25% glutaraldehyde at −90°C for 72 h. After warming over 9 h to −45°C and washing in dry acetone, samples were infiltrated at −20°C with LR White resin before polymerization under UV light at −20°C. Ultrathin sections were cut using a Reichert-Jung ultramicrotome and collected on formvar-coated gold grids.

### Immunofluorescence labelling

PnCcp was localized with rabbit PnCcp^Cpep^ or PnCcp^wp^ antibodies diluted 1:150 in 1% BSA and 0.1% fish skin gelatin (Sigma-Aldrich) in PBS. *Phytophthora* zoospore vesicle antigens PnLpv, PnVsv and PnCpa were localized with undiluted Lpv-1 and Vsv-1 monoclonal antibody supernatants and 10 µg/mL purified Cpa-2 monoclonal antibody, respectively (Hardham et al., 1994). *P. nicotianae* cyst wall protein was localized with undiluted Cpw-4 monoclonal antibody supernatant. All antibody incubations were at 37°C for 45 min and followed by rinsing in PBS. Secondary antibodies were fluorescein-conjugated sheep anti-mouse IgG (Jackson ImmunoResearch Laboratories, West Grove, PA, USA) diluted 1:150 or Alexa Fluor 488-GAR (Life Technologies, Carlsbad, CA, USA) diluted 1:1000. For double labelling, samples were incubated with PnCcp^Cpep^ or PnCcp^wp^ antibody at 4°C overnight followed by 45 min at 37°C with Alexa Fluor 488-GAR. Samples were then incubated with Lpv-1 followed by Texas Red-conjugated GAM (Jackson ImmunoResearch), each for 45 min at 37°C.

### Immunogold labelling

Sections were blocked in 1% BSA and 0.1% fish skin gelatin in PBS for 20 min then labelled with PnCcp^Cpep^ (diluted 1:600) or Lpv-1 (undiluted supernatant) antibodies, followed by GAM (GE Heathcare) or GAR (British BioCell) conjugated to 10 nm gold. For double immunogold labelling, sections were incubated in Lpv-1 and PnCcp^Cpep^ antibodies, followed by incubation in 5-nm gold-GAM (GE Healthcare) and 10-nm gold-conjugated GAR. Sections were counterstained with uranyl acetate and lead citrate before imaging (Hardham, 2001).

### Microscopy

Immunofluorescence was examined using an Axioplan epifluorescence microscope (Zeiss, Oberkochen, Germany) and photographed using MicroMax software with a black and white digital camera (Princeton Instruments, Trenton, NJ, USA). A Zeiss LSM780 confocal laser scanning microscope with a 1.4 NA 40× oil-immersion objective was used to examine *P. nicotianae* cells double-labelled with PnCcp^Cpep^ and Lpv-1 antibodies. Images were acquired using the Online Fingerprinting mode of Zen 2011 digital imaging software (Zeiss) in conjunction with multiphoton excitation at 810 nm (MaiTai eHP laser Spectra-Physics, Stahnsdorf, Germany). Laser group velocity was optimized using the MaiTai DeepSee attachment and associated software (Spectra-Physics). For ultrastructural studies, sections were examined with a Hitachi H7100FA transmission electron microscope.

### Enzyme-linked immunosorbent assay

*P. nicotianae* zoospore suspensions (∼10,000 zoospores per well) were loaded into 96-well filtration plates with 1.2 µm hydrophilic durapore membrane (Merck Millipore) on a vacuum manifold that facilitated rapid solution changes without loss of cells. Zoospore encystment was induced by addition of Ca(NO_3_)_2_ to a final concentration of 5 mM (Byrt, Irving & Grant, 1982) and cells fixed at selected time points thereafter in 4% formaldehyde and 0.2% glutaraldehyde in 50 mM PIPES buffer for 30 min. After washing in PBS and blocking in 3% BSA in PBS, primary antibodies diluted in PBS (PnCcp^Cpep^ at 1:150; Vsv-1 and Cpa-2 at 10 µg/mL; Cpw-4 undiluted supernatant) were added for 1 h at room temperature. After washing in PBT (0.5% v/v Tween 20 in PBS), samples were incubated in either sheep anti-mouse or sheep anti-rabbit IgG conjugated to horseradish peroxidase (Merck Millipore) diluted 1:2000 for 1 h at room temperature. Wells were washed in PBT before incubation in ABTS [2,2′-azino-bis(3-ethylbenzothiazoline-6-sulphonic acid)] substrate solution for 20 min. Absorbance at 405 nm was measured on a Multiskan RC microtiter plate reader (MTX Lab Systems, VA, USA). Three or four biological replicates were performed for each treatment and primary antibodies were omitted for controls.

### Dynamin inhibitor experiments

In dynamin inhibition experiments, the zoospore suspension was treated with 80 µM dynasore (Abcam Inc., Cambridge, MA, USA) in 0.8% dimethyl sulphoxide (DMSO) and immediately triggered to encyst with 5 mM Ca(NO_3_)_2_. In controls, zoospores were treated with 0.8% DMSO. For immunofluorescence labelling, the cells were fixed 2 min later in 4% formaldehyde as described above. The experiment was repeated four times. For membrane labelling of dynasore-treated cells, FM4-64 (Molecular Probes, Oregon, USA), to a final concentration of 1.5 µM, was added to the zoospore suspension treated with 80 µM dynasore (or 0.8% DMSO in controls). FM4-64 was added immediately after cells were triggered to encyst. Fluorescence was checked after 5 min. The experiment was repeated three times. For electron microscopy, zoospores were treated with 80 µM dynasore (or 0.8% DMSO in controls) and encystment immediately triggered with 5 mM Ca(NO_3_)_2_. Cysts were fixed in 1% glutaraldehyde in 100 mM PIPES buffer 5 min after induction of encystment and embedded in Lowicryl K4M as previously described (Hardham, 2001).

### Gene expression analysis

Expression of PnCcp and PnLpv genes was measured in three biological replicates by qPCR with *WS041* as the normalising gene as previously described (Gan et al., 2009). The 3′ end of PnLpv was cloned from *P. nicotianae* gDNA using degenerate primers designed against partial *P. cinnamomi* (NCBI accession AF315064) and *P. sojae* (Joint Genome Initiative accession jgi|Physo3|555774|estExt_Genewise1Plus.C_2_t20233) *Lpv* genes. Primers for PnCcp and PnLpv were ATGCTGCCACTTCTTCGC, CCGGAGTCGTGTTTGAGAAT, and AGGAAGAGGCTCGGGCTAAG, CTGGAAGTGCGGTGGCTG.

### Identification of dynamin homologs in *P. nicotianae*

Putative dynamins were identified by Pfam domain searches of proteins annotated by the *P. parasitica* INRA-310 Sequencing Project (http://www.broadinstitute.org/) and analysed with motif identification programs MyHits [http://myhits.isb-sib.ch/, (Pagni et al., 2007)], PROSITE [http://prosite.expasy.org/scanprosite/, (Sigrist et al., 2013)] and NCBI blastp [http://www.ncbi.nlm.nih.gov/, (Altschul et al., 1997)].

## Results

### Selective secretion of PnCcp from *P. nicotianae* zoospore large peripheral vesicles

Immunolabelling studies using a monoclonal antibody, Lpv-1, directed towards the high molecular weight glycoprotein, PcLpv, stored in the large peripheral vesicles in *P. cinnamomi* zoospores indicated that large peripheral vesicles do not undergo exocytosis during zoospore encystment but instead became dispersed throughout the cyst cytoplasm (Gubler & Hardham, 1988). Subsequent studies of another *Phytophthora* species, *P. nicotianae* (synonymous with *P. parasitica* var. *nicotianae*), revealed the presence of a second protein, PnCcp, within large peripheral vesicles (Škalamera & Hardham, 2006). Surprisingly, immunolabelling indicated that this protein was secreted from the large peripheral vesicles during zoospore encystment (Škalamera & Hardham, 2006).

**Figure 1:**
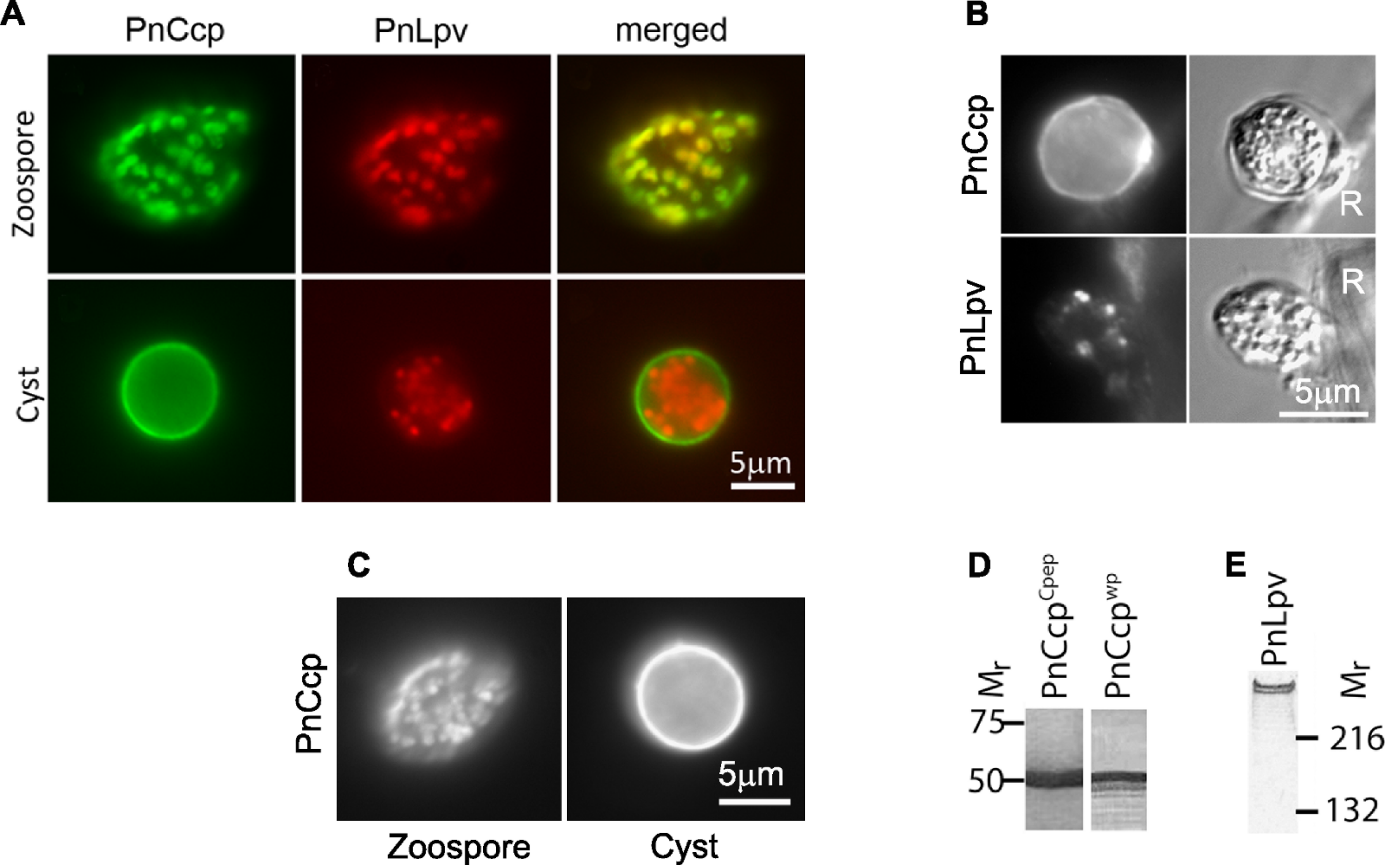
Immunolabelling of PnCcp and PnLpv proteins. (A) Double-immunofluorescence labelling of PnCcp (green) and PnLpv (red) proteins in *Phytophthora nicotianae* zoospores and cysts (10 min post-induction of encystment). In zoospores, PnCcp and PnLpv colocalize within large peripheral vesicles. During encystment, PnCcp, but not PnLpv, is secreted onto the cyst surface whereas PnLpv remains in large peripheral vesicles in the cyst cytoplasm. (B) Immunolabelling shows secretion of PnCcp during *P. nicotianae* zoospore encystment and attachment to the surface of *Lupinus angustfolius* roots (R). PnLpv proteins are retained within large peripheral vesicles in the cysts. (C) Immunolocalization of PnCcp using the PnCcp^wp^ antibody raised against the full-length PnCcp protein in *P. nicotianae*. PnCcp^wp^ labels zoospore large peripheral vesicles and the cyst surface. (D) Immunoblot assay of purified PnCcp fusion protein coupled to maltose binding protein (MBP) labelled with PnCcp^Cpep^ and PnCcp^wp^ antibodies. The *M_r_* of 53 kDa of the labelled polypeptide corresponds to the predicted size of PnCcp-MBP fusion protein. (E) Immunoblot assay of *P. nicotianae* proteins labelled with Lpv-1 antibody which reacts with two PnLpv isoforms with a *M_r_* > 400 kDa.

We have performed immunofluorescence double-labelling of *P. nicotianae* zoospores and cysts using the PnCcp polyclonal antibody which was raised against a C-terminal peptide (hereafter designated, PnCcp^Cpep^ antibody) and the Lpv-1 monoclonal antibody to re-assess the initial observations. In zoospores, PnCcp^Cpep^ and Lpv-1 antibodies label the same vesicles within the zoospore cortex (Fig. 1A). However, in young cysts, PnCcp^Cpep^ antibody labels the cyst surface, whereas Lpv-1 antibody continues to label the large peripheral vesicles within the cyst cytoplasm (Fig. 1A). These results indicate that PnCcp and PnLpv are both stored in the large peripheral vesicles in zoospores and that during zoospore encystment, PnCcp is secreted from the vesicles while PnLpv is not.

The experiments described above were conducted *in vitro*, with zoospore encystment induced by vortexing. To investigate whether selective secretion of PnCcp also occurred during plant infection, lupin (*Lupinus angustifolius*) roots were inoculated with *P. nicotianae* zoospores before being fixed and immunofluorescently labelled with PnCcp^Cpep^ antibody. Labelling of PnCcp proteins was detected on the surface of the cysts attached to the root surface (Fig. 1B). No labelling of PnLpv proteins was observed on the surface of the cysts that were attached to the plant roots (Fig. 1B).

### Verification of the specificity of the PnCcp^Cpep^ antibody

The PnCcp^Cpep^ polyclonal antibody was raised against a 15-amino acid synthetic peptide from the C-terminus of the PnCcp protein (Škalamera & Hardham, 2006). To verify the specificity of the PnCcp^Cpep^ antibody, the full length of the PnCcp protein (lacking the signal peptide) was expressed as a recombinant protein in *E. coli*, purified and used to raise a new polyclonal antiserum (designated PnCcp^wp^ for “whole protein”). Immunofluorescence labelling with the PnCcp^wp^ antiserum showed the same localization as that obtained with the PnCcp^Cpep^ antibody. PnCcp^wp^ antibodies labelled the large peripheral vesicles in zoospores and the surface of cysts (Fig. 1C). When the PnCcp^Cpep^ and PnCcp^wp^ antibodies were tested on immunoblots containing PnCcp fusion protein purified from *E. coli*, both antibodies reacted with a single polypeptide with a relative molecular weight of 53 kDa (Fig. 1D). This size was the same as that predicted for the recombinant PnCcp protein which was fused to the maltose binding protein (MBP). Pre-immune sera displayed no labelling in immunofluorescence or immunoblot assays when used at the same concentrations as the immune sera. Neither antiserum labelled immunoblots of proteins extracted from *P. nicotianae* cells, possibly because of the small size of the PnCcp protein. In immunoblots, the Lpv-1 monoclonal antibody reacts with two polypeptides of relative molecular mass greater than 400 kDa (Fig. 1E).

### Timing of secretion of PnCcp

The large peripheral vesicles move away from the plasma membrane about 5 min after commencement of zoospore encystment (Gubler & Hardham, 1988). In order to determine whether the secretion of PnCcp occurs while the large peripheral vesicles are close to the plasma membrane or after their migration out of the cell cortex, we investigated the timing of PnCcp secretion by immunofluorescence labelling and enzyme-linked immunosorbent assays (ELISAs). As well as PnCcp^Cpep^ and Lpv-1 antibodies, monoclonal antibodies Vsv-1, Cpa-2 and Cpw-4 (Hardham et al., 1994) were also used to allow comparison of the relative timing of exocytosis of PnCcp with that of PnVsv and PnCpa proteins from ventral and dorsal vesicles, respectively, and of a cyst wall protein, PnCpw. In these experiments, *P. nicotianae* zoospores were induced to encyst by the addition of Ca(NO_3_)_2_ and samples taken at intervals during the first 5 min of the encystment process. Zoospores were chemically fixed using a formaldehyde fixative that does not preserve the zoospore plasma membrane and which thus allows antibodies to react with intracellular proteins. Encysting cells were fixed with a fixative that contained glutaraldehyde in addition to formaldehyde. This fixative preserves the spore plasma membrane which prevents antibody access to intracellular proteins (Hardham, 1985). This ensured that the antibodies labelled only material that had been secreted onto the cell surface in the samples fixed after induction of encystment.

**Figure 2:**
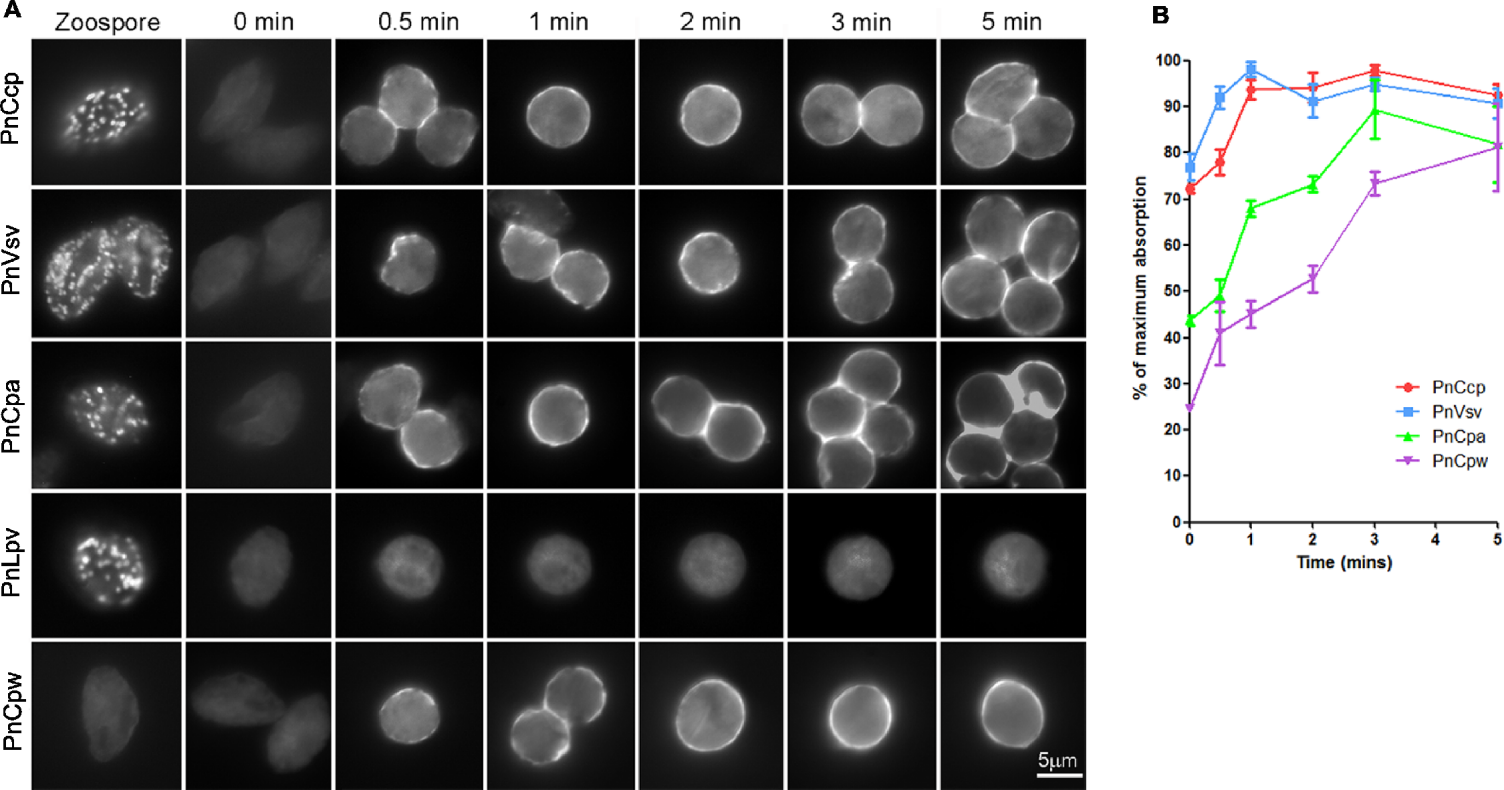
Immunofluorescence labelling of zoospores and encysting cells. (A) Fixation of zoospores allowed intracellular labelling but fixation of 0–5 min encysting cells allowed only cell surface labelling. PnCcp (labelled with PnCcp^Cpep^), PnVsv and PnCpa proteins are stored in cortical vesicles in the zoospores and are secreted rapidly during spore encystment. PnLpv protein is also stored in zoospore peripheral vesicles but is not secreted. PnCpw, a cyst wall-associated protein, accumulates progressively on the cyst surface. (B) ELISA quantification of protein secretion. PnCcp (red circles), PnVsv (blue squares), PnCpa (green triangles) and PnCpw (purple inverted triangles), are released during the first 5 min after induction of zoospore encystment. Data are expressed as a percentage of the maximum absorbance for each antibody. Because of technical constraints, “zero” min points are ∼10 s post-induction of encystment. Bars indicate s.e.m. (*n* = 3).

The immunofluorescence assay revealed that 30 s after the induction of encystment, labelling of PnCcp could be observed on some regions but not the entire surface of encysting spores (Fig. 2A). Labelling of ventral vesicle, dorsal vesicle and cyst wall proteins with Vsv-1, Cpa-2 and Cpw-4, respectively, was also detected on the cyst surface 30 s after induction of encystment (Fig. 2A). This suggested that secretion of PnCcp from the large peripheral vesicles begins at a similar time to that of the secretion of ventral and dorsal vesicle proteins and the cyst wall protein. As encystment continued, labelling of PnCcp appeared stronger and 2 min after encystment induction, the entire surface of most cysts was labelled by PnCcp^Cpep^ antibody. Labelling by Vsv-1, Cpa-2 and Cpw-4 showed a similar temporal and spatial pattern. No secretion of PnLpv proteins was detected at any time point after encystment was induced (Fig. 2A).

An ELISA was used to quantify the timing of PnCcp protein secretion onto the surface of spores during zoospore encystment (Fig. 2B). As in the immunofluorescence assay, the encysting cells were fixed so that only surface proteins were labelled. Because of an unavoidable delay between the addition of Ca^2+^ to induce encystment and addition of fixative, values at 0 min are about 10–15 s post-induction. The results show that secretion of PnCcp, PnVsv, PnCpa and PnCpw proteins begins within 30–60 s after encystment was induced. When values are expressed as a percentage of the maximum absorbance, the results show that PnVsv is fully secreted and over 90% of PnCcp is secreted by 1 min post-induction. By contrast, PnCpa is not fully secreted until 3 min post-induction and PnCpw secretion increases progressively over the 5-min time period. Importantly, the results indicate that secretion of PnCcp proteins within 1–2 min of the onset of encystment occurs when the large peripheral vesicles are still in the zoospore cortex.

### Synthesis of PnCcp and PnLpv proteins

Earlier studies suggested that large peripheral vesicles are absent from vegetative hyphae, appear during sporulation and sometimes contain only PnLpv or only PnCcp (Dearnaley, Maleszka & Hardham, 1996; Škalamera & Hardham, 2006). A possible explanation for the latter observation could be that the two proteins are synthesized at different locations or at different times and are subsequently brought together by vesicle fusion. Growing fungal and Oomycete mycelial colonies have a distinct polarity, with young cells extending by tip-growth predominantly at the colony’s margin and progressively older hyphae occurring towards the colony’s centre. To investigate the relative timing of the synthesis of PnCcp and PnLpv, the occurrence of vesicles containing these proteins in *P. nicotianae* colonies growing on V8 agar plates was determined using immunofluorescence double-labelling with PnCcp^Cpep^ and Lpv-1 antibodies. Samples were taken at 5 mm intervals across the colony, thus representing a time course of development from old to young hyphae.

**Figure 3:**
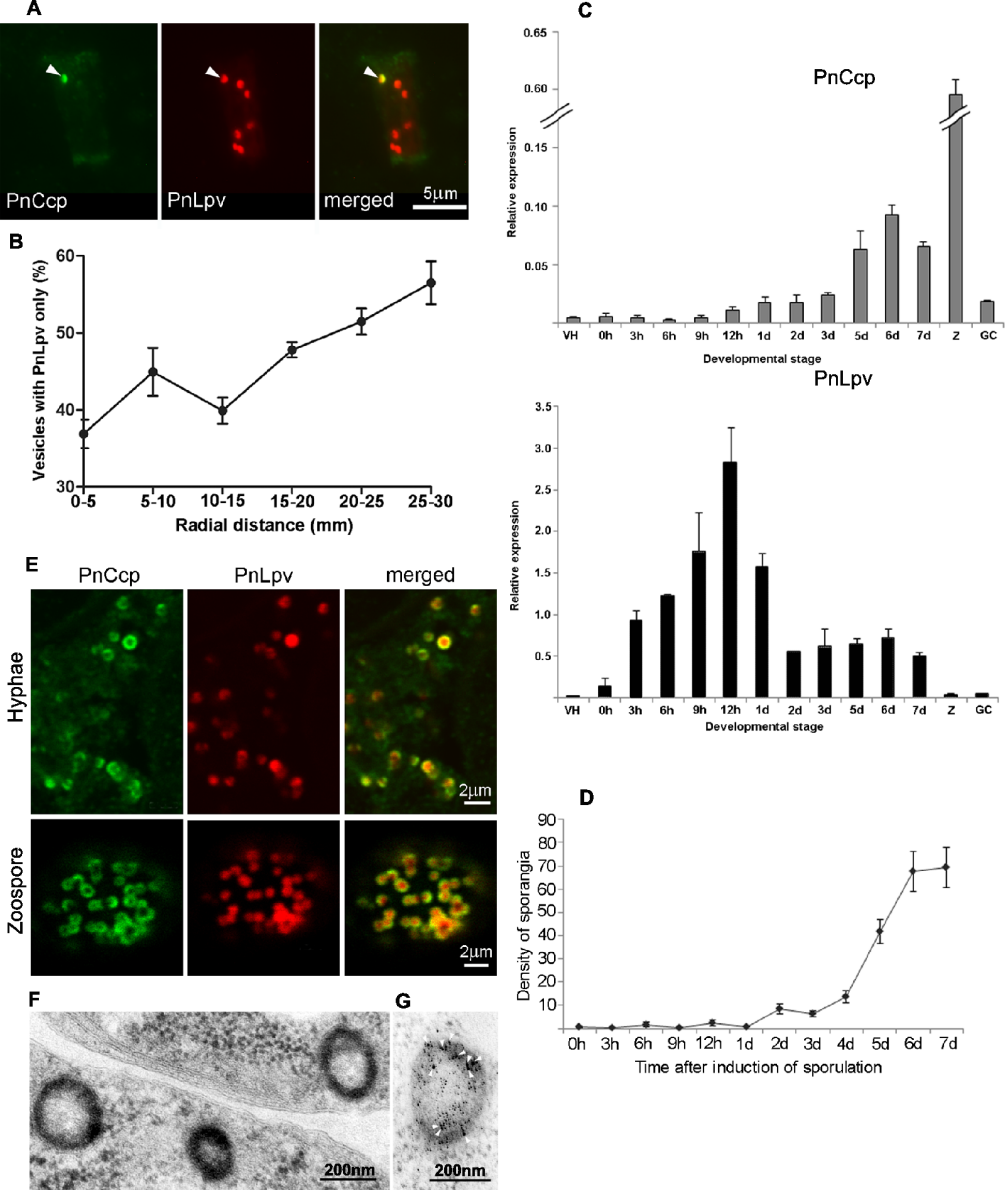
PnCcp and PnLpv synthesis and packaging into large peripheral vesicles. (A) Double-immunofluorescence labelling with PnCcp^Cpep^ and Lpv-1 antibodies shows only one (arrowhead) of seven vesicles in a hyphal fragment from the leading edge of mycelium growing on nutrient agar contains PnCcp in addition to PnLpv proteins. (B) Quantitation of vesicles that contain PnLpv only in hyphae sampled at 5-mm intervals across a mycelial colony from its centre (0–5 mm) to the advancing edge (25–30 mm). Bars indicate s.e.m. (*n* = 3). (C) qPCR quantitation of PnCcp and PnLpv transcript levels in vegetative hyphae (VH), sporulating hyphae (0 h to 7 days), zoospores (Z) and 3-h germinated cysts (GH). Expression levels are relative to the normalising gene, *WS041*. Bars indicate s.e.m. (*n* = 3). (D) Density of sporangia (per microscope field of view) in *P. nicotianae* mycelia growing in liquid culture after induction of sporulation. Bars show s.e.m. (*n* = 3). (E) Double-immunofluorescence labelling with PnCcp^Cpep^ and Lpv-1 antibodies of *P. nicotianae* sporulating hyphae and zoospores. Confocal microscope optical sections show that PnCcp (green) is often restricted to an outer zone within the large peripheral vesicles while PnLpv (red) occurs throughout the vesicles. (F, G) Large peripheral vesicles in cleaving sporangia of *P. nicotianae* prepared by plunge-freezing and freeze-substitution. Ultrastructural analysis reveals an outer shell of electron-dense material (F) which is labelled by PnCcp^Cpep^-goat anti-rabbit-10 nm gold (arrowheads). Lpv-1-goat anti-mouse-5 nm gold labels throughout the vesicle (G).

Analysis of the hyphal samples did not identify any subcellular components that were labelled only by the PnCcp^Cpep^ antibody, however, it did reveal the existence of two populations of vesicles: one category of vesicle contained both PnCcp and PnLpv proteins whereas the other category contained PnLpv only (Fig. 3A). The occurrence of these two categories of vesicle was quantified along the age gradient across the mycelial colony and the results showed that the percentage of vesicles that contained only PnLpv increased from 37% at the centre of the colony to 56% at the growing edge of the colony (Fig. 3B). These data suggest that the synthesis of PnLpv precedes that of PnCcp in hyphae at the advancing edge of the colony, that when at least a proportion of the vesicles are first made they contain only PnLpv and that PnCcp is subsequently added to the PnLpv-containing vesicles.

### Timing of expression of PnCcp and PnLpv genes

The relative timing of production of PnCcp and PnLpv was also investigated by determining the patterns of expression of PnCcp and PnLpv genes using real-time quantitative RT-PCR (qPCR). The expression of both genes was up-regulated during sporulation but maximum expression occurred at different times after induction of sporulation (Fig. 3C). Both genes were expressed at extremely low levels in vegetative hyphae. After induction of sporulation, PnCcp transcript levels increased slowly, peaking 6 d after induction. In contrast, PnLpv transcript levels increased quickly, peaking 12 h after induction of sporulation. The highest level of PnCcp transcripts detected occurred when the density of sporangia reached a maximum level (Fig. 3D). Analysis of other stages of the *P. nicotianae* asexual life-cycle showed that PnCcp was most highly expressed in zoospores and that PnLpv was most highly expressed in 12-h sporulating hyphae (Fig. 3C). The qPCR results thus provide evidence of the differential expression of PnCcp and PnLpv genes, with synthesis of PnLpv transcripts occurring much earlier than that of PnCcp during sporulation.

### Compartmentalization of PnCcp in large peripheral vesicles

In order to further investigate details of the formation and function of large peripheral vesicles, we determined whether there was any evidence of differential distribution of PnCcp and PnLpv within the vesicles. Localization of PnCcp and PnLpv within individual vesicles was studied by immunofluorescence double-labelling and two-colour confocal microscopy and by immunogold transmission electron microscopy.

For confocal microscopy, the ability of multiphoton excitation to simultaneously excite multiple fluorochromes in a small focal volume was exploited to avoid chromatic aberration that may occur during multi-wavelength scans in single-photon excitation. Online Fingerprinting was utilised to remove both non-specific fluorescence and to accurately compensate for any fluorescent bleed-through between Alexa Fluor 488 and Texas Red. Reference spectra used for Online Fingerprinting were derived from singly-stained hyphae and zoospores, using the same preparation and optical configuration as the two-colour experiments. Vesicles in *P. nicotianae* sporulating hyphae and zoospores were observed (Fig. 3E). While Lpv-1 labelling of PnLpv occurred throughout the vesicles, in many cases PnCcp occupied only part of the vesicle. In 84% of the large peripheral vesicles examined (data from 1049 vesicles in 16 zoospores), PnCcp was confined to an outer region within the vesicle.

The compartmentalization of PnCcp was also examined ultrastructurally using immunogold single- and double-labelling. In chemically-fixed material, the contents of large peripheral vesicles appear homogeneous, and immunogold labelling with PnCcp^Cpep^ and Lpv-1 antibodies usually occurs throughout the vesicle (Škalamera & Hardham, 2006). In some cases, however, there was evidence of peripheral labelling of PnCcp within the vesicles. To minimize artifacts that might be generated during chemical fixation, *P. nicotianae* sporulating hyphae were prepared for immunogold labelling by plunge-freezing and freeze-substitution. Electron microscopy of ultrathin sections of cryopreserved *P. nicotianae* sporangia revealed an electron-dense peripheral ring within the large peripheral vesicles (Fig. 3F). Immunogold double-labelling showed that PnCcp^Cpep^ antibody consistently labelled the electron-dense outer region of the vesicles whereas labelling with the Lpv-1 antibody occurred throughout the vesicle (Fig. 3G).

### Role of dynamin in selective secretion of PnCcp

The immunofluorescence and ELISA experiments described above show that PnCcp is secreted soon after induction of zoospore encystment when the large peripheral vesicles are in close proximity to the zoospore plasma membrane. An existing model of secretion that could explain the release of PnCcp and retention of PnLpv is transient fusion, also called kiss-and-run secretion. In this model, a narrow fusion pore forms between a secretory vesicle and the plasma membrane, allowing the release of some but not all of the vesicle contents before the pore reseals and the vesicle pinches away from the plasma membrane (Harata, Aravanis & Tsien, 2006; van Kempen et al., 2011). Dynamin, a GTPase associated with the budding and scission of newly formed vesicles in endocytosis (Chappie et al., 2011), has been reported to also play an important role in kiss-and-run secretion in mammalian cells, being involved in severing the neck of the fusion pore and enabling the vesicle to pinch away from the plasma membrane (Jaiswal, Rivera & Simon, 2009). Bioinformatic analysis of the *P. nicotianae* genome revealed the presence of six predicted genes that encode proteins containing from one to three dynamin-like domains. The six genes have little overall homology. One gene, PPTG_15137, encodes a predicted protein that contains the GTPase domain, the middle domain and the GTPase effector domain typical of dynamin-like proteins. It shares 49% amino acid identity with the human dynamin-1-like protein isoform 2 (NCBI accesssion NP_036193) and 95% identity to a dynamin-like protein characterised in *P. sojae* (Li et al., 2013). PPTG_15137 is highly expressed during the infection of lupin roots (LM Blackman and AR Hardham, unpublished observations). To investigate whether the selective secretion of PnCcp is due to kiss-and-run secretion, we determined the role of dynamin in large peripheral vesicle dynamics by treating living *P. nicotianae* zoospores with dynasore, a cell-permeant inhibitor of the dynamin GTPase (Macia et al., 2006; Jaiswal, Rivera & Simon, 2009).

**Figure 4:**
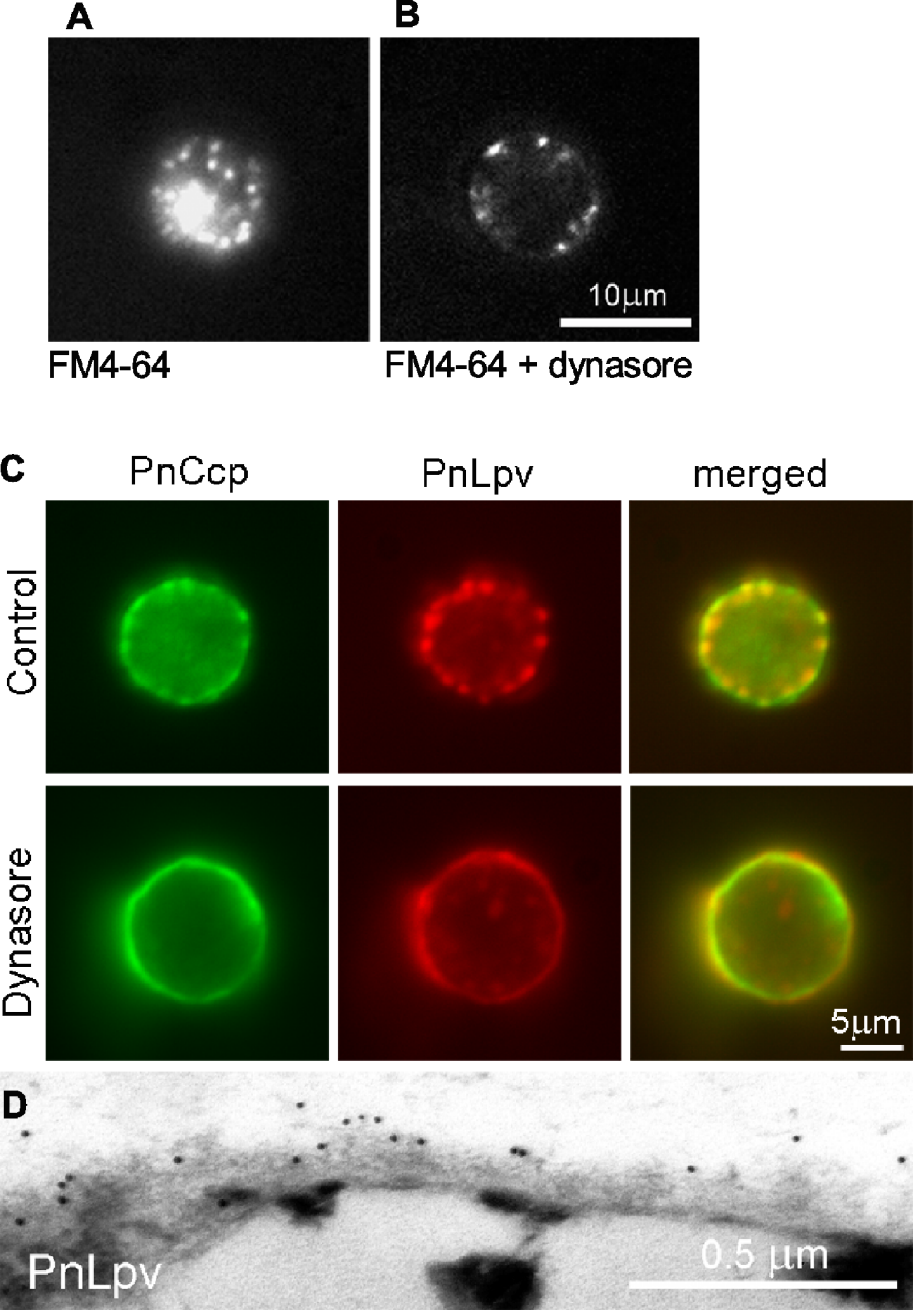
The effect of the dynamin inhibitor, dynasore, on selective secretion of PnCcp. (A, B) Uptake of FM4-64-labelled plasma membrane in *P. nicotianae* zoospores 5 min after induction of zoospore encystment. (A) In control zoospores incubated in FM4-64, endocytosis of the zoospore plasma membrane during encystment leads to high levels of cytoplasmic fluorescence within the spores. (B) In zoospores incubated in FM4-64 and 80 µM dynasore, no plasma membrane internalization occurs and FM4-64-labelled membranes remain at the cell surface. (C) Double-immunofluorescence labelling with PnCcp^Cpep^ and Lpv-1 antibodies of *P. nicotianae* cells incubated in 0.8% DMSO (control) or 80 µM dynasore and fixed 2 min after the induction of encystment. Treatment with 80 µM dynasore results in secretion of PnLpv proteins from the large peripheral vesicles. (D) Lpv-1 immunogold labelling shows PnLpv proteins on the surface of cyst fixed 5 min after the induction of encystment.

Because regulated secretion of PnCcp occurs within 1–2 min after the induction of encystment, it was necessary that dynasore was able to act rapidly to inhibit dynamin in the encysting spores. To test this, the ability of dynasore to inhibit endocytosis during zoospore encystment was investigated by labelling the zoospore plasma membrane with FM4-64. FM4-64 dye is widely used for tracking endocytosis as it can intercalate into the plasma membrane and be taken into the cells by endocytosis (Vida & Emr, 1995). The experiments showed that in *P. nicotianae* zoospores treated with FM4-64 and 80 µM dynasore for 5 min, the amount of FM4-64-labelled membrane that was internalised into the cytoplasm was much less than in control cells (Figs. 4A and 4B). FM4-64-labelled vesicle-like structures that formed in the presence of dynasore remained at the plasma membrane, possibly indicating that inhibition of dynamin function may have prevented invaginated membranes from undergoing fission from the plasma membrane. Importantly, the results confirmed that dynasore could block the activity of dynamin in the short time-frame necessary for the experiments with living zoospores.

To monitor the secretion of proteins from the large peripheral vesicles, cells treated with 80 µM dynasore were fixed 2 min after inducing encystment and immunofluorescently double-labelled with PnCcp^Cpep^ and Lpv-1 antibodies. The fixation regime allowed both internal and surface labelling. In control cells treated with the solvent 0.8% DMSO alone, PnCcp, but not PnLpv, was secreted onto the spore surface as normal (Fig. 4C). In cells treated with dynasore, PnCcp was secreted as usual but in addition, PnLpv was also secreted onto the surface of the spores and there were no longer any large peripheral vesicles containing PnLpv in the spore cytoplasm (Fig. 4C). Dynasore treatment appeared to slightly increase the speed of PnCcp secretion. In most control cells in 2-min samples (either untreated or treated with 0.8% DMSO), PnCcp occurred both in intracellular vesicles and on the cell surface (Fig. 4C), indicating that secretion of PnCcp was in process but not complete. By contrast, in the dynasore-treated, 2-min samples, there was no PnCcp in intracellular vesicles and all PnCcp had been secreted (Fig. 4C). In samples treated with dynasore, the cells were slightly larger than control cells, a feature that could be due to inhibition of endocytic plasma membrane retrieval during encystment.

Secretion of PnLpv in the dynasore-treated cysts was confirmed by immunogold labelling of ultrathin sections with the Lpv-1 antibody. Dynasore-treated samples showed a reduction in the number of large peripheral vesicles in the cysts. In contrast to the situation in control cells, where there is no Lpv-1 labelling of the cell surface, in dynasore-treated cysts, PnLpv labelling was seen outside the cyst cell wall (Fig. 4D). Treatment with 80 µM dynasore had only a small effect on cell viability. On removing the dynasore after a 2 min treatment, 87.3% of the cysts germinated compared to 93% in controls. An interpretation of the dynasore experiments is that large peripheral vesicles normally undergo kiss-and-run secretion but when dynamin activity is inhibited by dynasore, the fusion pore formed is unable to reseal and all the vesicle contents, including both PnCcp and PnLpv, are secreted.

## Discussion

Protein secretion is a fundamental process in cell life and is critical for the establishment of infection by bacterial and eukaryotic pathogens of both plants and animals. Protein secretion required for the initiation of infection is triggered by host factors perceived by the pathogen after its arrival at the host surface. In the case of fungal and Oomycete pathogens of plants, both physical and chemical host signals trigger regulated secretion including that of adhesives and degradative enzymes, however, little is known about the molecular mechanisms regulating protein secretion in these organisms. The regulated secretion that occurs during attachment of spores of the plant pathogen *P. nicotianae* to a potential host involves an unusual process whereby a protein is selectively secreted from vesicles that remain otherwise intact. In the research reported in this paper, the selective secretion of the Sushi domain-containing protein, PnCcp, has been analyzed using immunolabelling and pharmacological experiments. The results reveal that selective secretion of PnCcp occurs through the transient fusion of the large peripheral vesicles. To the best of our knowledge, selective protein secretion via transient fusion has not heretofore been demonstrated outside mammalian cells.

### A transient fusion mechanism underlies selective secretion of PnCcp

Secretion of PnCcp and retention of PnLpv within large peripheral vesicles during encystment of *P. nicotianae* zoospores could be explained by two existing models of protein trafficking. One is the “sorting-by-retention” model in which some proteins are retained within a vesicle, possibly due to their aggregated state, while others are removed by recruitment into clathrin-coated vesicles and trafficked to endomembrane compartments or the cell surface (Arvan & Castle, 1998; Arvan & Halban, 2004; Robinson, Oliviusson & Hinz, 2005).

Application of the sorting-by-retention model predicts that PnCcp would be specifically removed from the large peripheral vesicles in small vesicles, leaving behind other proteins, including PnLpv. These PnCcp-containing vesicles would then fuse with the zoospore plasma membrane during encystment, thus releasing PnCcp onto the spore surface. According to this model, the membrane of the large peripheral vesicle would at no time fuse with the plasma membrane. Two observations suggest that this model does not apply to PnCcp secretion. Firstly, small PnCcp-transporting vesicles in the cortex of encysting spores have not been seen. Secondly, dynasore inhibition of dynamin function in pore closure leads to PnLpv secretion, indicating that the large peripheral vesicles do indeed fuse with the spore plasma membrane.

The alternative model that could explain selective secretion of PnCcp is transient fusion, also called kiss-and-run secretion. According to this model, some vesicle cargo proteins are released through a narrow fusion pore that forms transiently between vesicle and plasma membranes (Alabi & Tsien, 2013). This process has been documented in a number of mammalian cell types. In adrenal chromaffin cells, for example, transient fusion allows dense-core vesicles to selectively release small soluble catecholamines but retain less mobile neuropeptides (Fulop, Radabaugh & Smith, 2005; Zhang et al., 2011). In pancreatic beta cells, transient fusion allows secretory granules to selectively release small transmitters but retain insulin peptides (Obermüller et al., 2005; MacDonald et al., 2006). In endothelial cells, transient fusion allows Weibel-Palade bodies to release proinflammatory cytokines while retaining core proteins (Babich et al., 2008).

Two lines of evidence indicate that transient fusion is the likely mechanism operating during selective secretion of PnCcp from the large peripheral vesicles in *P. nicotianae* zoospores. Firstly, our immunofluorescence labelling and ELISAs show that PnCcp is secreted 1–2 min after the induction of encystment and thus occurs while the large peripheral vesicles are closely apposed to the spore plasma membrane (Gubler & Hardham, 1990; Škalamera & Hardham, 2006). Secondly, when dynamin GTPase activity is inhibited by dynasore, PnCcp and PnLpv are both released from the large peripheral vesicles and the number of PnLpv-containing vesicles remaining after encystment is greatly reduced. These experimental results are consistent with dynasore-induced full fusion of large peripheral vesicles with the plasma membrane and complete release of all vesicle cargo molecules. Dynamin forms a constricting collar around the neck of clathrin-coated pits, and facilitates fission of the membrane connection between vesicles and the plasma membrane during endocytosis and kiss-and-run secretion (Tsuboi, McMahon & Rutter, 2004; Jaiswal, Rivera & Simon, 2009; Chappie et al., 2011). In our dynasore experiments, the rate of PnCcp release was slightly faster than usual. Inhibition of dynamin activity in mammalian fibrosarcoma cells that normally utilize transient fusion, also results in full fusion and more rapid release of cargo molecules than occurs during transient fusion (Jaiswal, Rivera & Simon, 2009). Taken together, these data support the proposal that selective secretion of PnCcp is achieved through the transient fusion of large peripheral vesicles in encysting zoospores.

### Factors underlying selective secretion of PnCcp

What factors could be responsible for secretion of PnCcp and retention of PnLpv during transient fusion of large peripheral vesicles during *P. nicotianae* zoospore encystment? A prime candidate from other studies is the size of the cargo molecules. Typically, small soluble molecules are secreted while large less soluble molecules are retained (Fulop, Radabaugh & Smith, 2005; Obermüller et al., 2005; MacDonald et al., 2006; Babich et al., 2008; Zhang et al., 2011). Discrimination on the basis of molecule size is achieved through limitations in the size of the transient fusion pore. Selective secretion of PnCcp, a 10 kDa protein, and retention of PnLpv, a >400 kDa glycoprotein, from large peripheral vesicles in *Phytophthora* zoospores provides another example of this principle.

Our research on secretion in *Phytophthora* zoospores highlights another factor potentially involved in selective secretion, namely subcompartmentalization of vesicle components. Ultrastructural examination and immunocytochemical labeling show that PnCcp is restricted to an outer electron-dense zone within the large peripheral vesicles in the *Phytophthora* zoospores.

Heterogeneity in the appearance of vesicle contents is not unusual but demonstration of subcompartmentalization of specific vesicle cargo molecules is less common. Notable examples of sub-organelle molecular compartmentation include rhoptries in malaria zoites and dense-core vesicles in pancreatic beta cells (Obermüller et al., 2005; Kats et al., 2006; Boothroyd & Dubremetz, 2008; Suckale & Solimena, 2010). In malarial zoites, proteins may be confined to the rhoptry tip, neck, bulb or base/periphery (Zuccala et al., 2012). In pancreatic beta cells, tightly-packed insulin crystals form an electron-dense core that is surrounded by a halo of small soluble molecules in the dense-core vesicles. These two examples provide evidence of the importance of sub-vesicle compartmentalization in regulation of cargo secretion. In malarial parasites, subcompartmentalization of rhoptry proteins determines the order in which the proteins are released during host cell invasion (Zuccala et al., 2012). In pancreatic beta cells, subcompartmentalization within dense-core vesicles is associated with differences in the timing of cargo release during full fusion; the molecules in the outer zone of the vesicles are released before insulin peptides are released from the central crystalline core (Obermüller et al., 2005).

We thus conclude that selectivity of protein secretion from *Phytophthora* large peripheral vesicles could be achieved by limitations of fusion pore diameter so that only small proteins like PnCcp are released, by pore kinetics, by cargo subcompartmentalization during vesicle biogenesis, or by a combination of these factors. It remains for future studies to determine their relative importance, however, our quantification of the co-existence of both proteins within vesicles during development may provide clues as to how the subcompartmentation is generated. More than 50% of vesicles contain only PnLpv in young hyphae but this value decreases as hyphae age and in zoospores all large peripheral vesicles contain both proteins. The data suggest that PnCcp is added after PnLpv-containing vesicles have formed. High levels of PnCcp gene expression in zoospores could reflect continued addition of PnCcp proteins in spores. Subsequent addition of a protein to an existing vesicle is not without precedent. In malaria zoites, three rhoptry proteins that are synthesized early and stored in the ER and Golgi apparatus are translocated to the rhoptries once they have formed (Sam-Yellowe, Shio & Perkins, 1988; Topolska et al., 2004; Siddiqui et al., 2013).

Given their different fates, why is PnCcp not packaged separately from PnLpv? One possible explanation is that it is a consequence of organelle allocation mechanisms operating during sporangial cleavage and zoosporogenesis. During sporangial cleavage, the three categories of peripheral vesicles are translocated to specific regions of the zoospore plasma membrane as it develops (Hyde et al., 1991). There may be constraints on the number of vesicle types that can be accurately deployed between elements of the peripheral cisternae (Hardham, 1987), a membranous system similar to the inner membrane complex in apicomplexans (Kono et al., 2012).

### Concluding remarks

This study of *Phytophthora* zoospores presents evidence that transient fusion of one category of cortical vesicle provides a mechanism for selective protein secretion during the initial phase of host infection. This is the first demonstration of transient fusion in the Oomycetes and, we believe, it is the first time that differential secretion of vesicle contents has been shown outside the animal kingdom. Although patch-clamping experiments suggest that transient fusion may occur in plant protoplasts (Weise et al., 2000; Thiel, Kreft & Zorec, 2009), regulation of protein secretion via this mechanism has not been shown in plants. The cyclical discharge of the water-expulsion vacuole in *Dictyostelium discoideum* has been viewed as a form of kiss-and-run secretion (Essid et al., 2012), however, the size and dynamics of the pore that forms between contractile vacuole bladder and plasma membrane are likely to be quite different to those of pores forming during vesicular kiss-and-run secretion; nor is there any evidence of selectivity in vacuole content release. In malarial cells, the sequential release of rhoptry proteins does not involve transient fusion (Zuccala et al., 2012).

During *Phytophthora* zoospore encystment, full fusion of dorsal and ventral vesicles and transient fusion of large peripheral vesicles are induced simultaneously by the same signal, be it Ca^2+^ uptake, ligand binding to the flagella or mechanical stimulation (Byrt, Irving & Grant, 1982; Hardham & Suzaki, 1986). *Phytophthora* zoospores thus constitute a system in which controls of the form of regulated secretion may be investigated within co-existing vesicle populations. In terms of zoospore physiology, selective secretion of PnCcp may be part of the process of spore attachment to the host surface, as Sushi domain-containing proteins are often involved in adhesion and protein-protein interactions. PnLpv, on the other hand, is a storage protein used to support early germling growth before host-derived nutrients are accessible (Gubler & Hardham, 1990). Further studies of factors that may regulate secretion during zoospore encystment promise to increase our understanding of transient fusion and selective protein secretion not only in *Phytophthora* but also in other cells.

**Figure 5:**
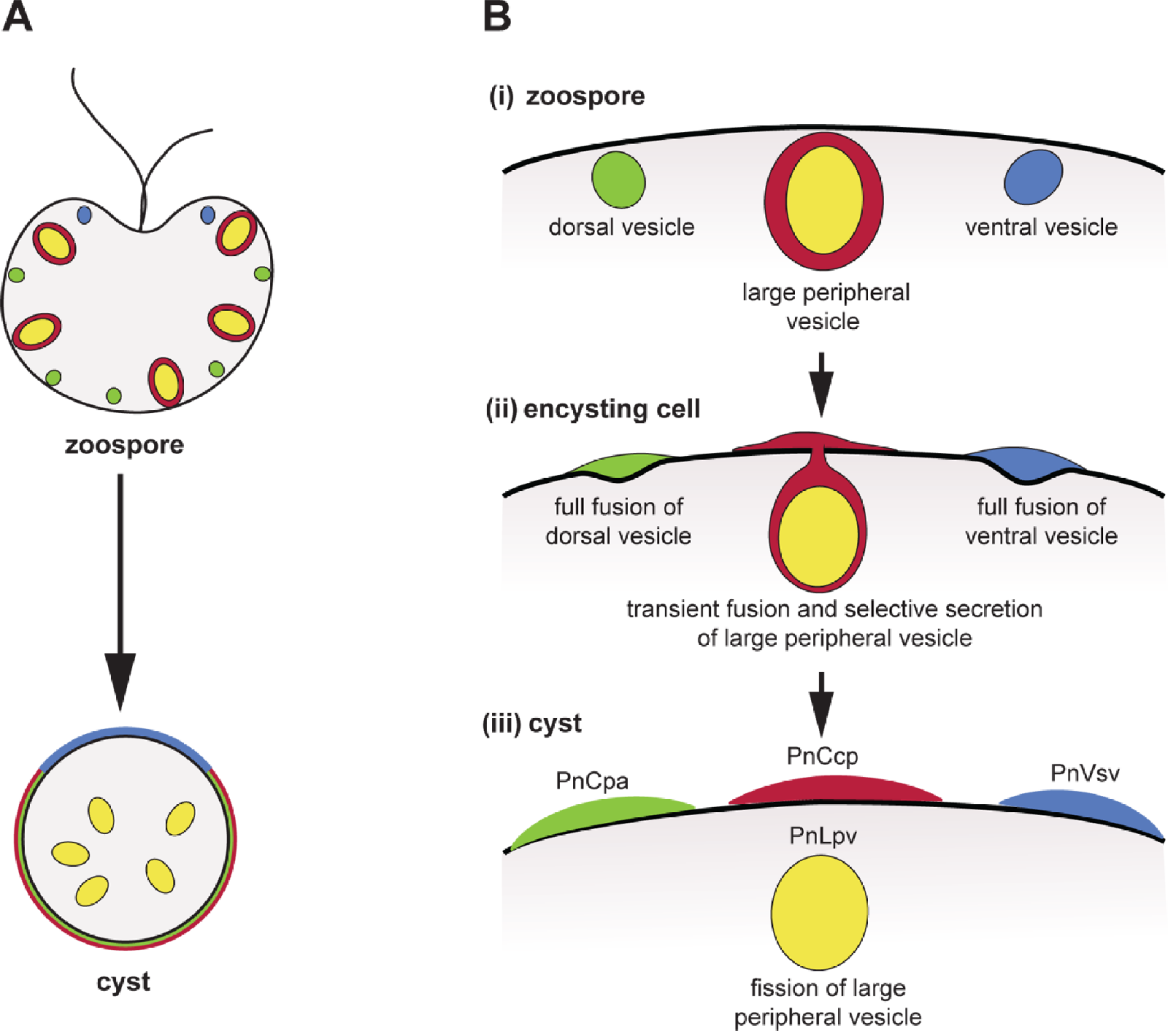
Co-induction of full fusion and transient fusion of *Phytophthora* zoospores vesicles during encystment. (A) Localization of dorsal (green), large peripheral (red-yellow) and ventral (blue) vesicles and their contents within zoospores and a young cyst. (B) Behaviour of the three types of peripheral vesicles (i) before, (ii) during and (iii) after induction of regulated secretion. Dorsal (green) and ventral (blue) vesicles undergo full fusion, secreting their entire contents onto the spore surface. Large peripheral vesicles (red-yellow) undergo transient fusion during which PnCcp proteins (red) are selectively secreted and PnLpv (yellow) proteins are retained. After fission of the fusion pore, large peripheral vesicles move away from the plasma membrane.

## References

[ref-1] Alabi AA, Tsien RW (2013). Perspectives on kiss-and-run: role in exocytosis, endocytosis, and neurotransmission. Annual Review of Physiology.

[ref-2] Altschul SF, Madden TL, Schäffer AA, Zhang J, Zhang Z, Miller W, Lipman DJ (1997). Gapped BLAST and PSI-BLAST: a new generation of protein database search programs. Nucleic Acids Research.

[ref-3] Arvan P, Castle D (1998). Sorting and storage during secretory granule biogenesis: looking backward and looking forward. Biochemical Journal.

[ref-4] Arvan P, Halban PA (2004). Sorting ourselves out: seeking consensus on trafficking in the beta-cell. Traffic.

[ref-5] Babich V, Meli A, Knipe L, Dempster JE, Skehel P, Hannah MJ, Carter T (2008). Selective release of molecules from Weibel-Palade bodies during a lingering kiss. Blood.

[ref-6] Bandmann V, Kreft M, Homann U (2011). Modes of exocytotic and endocytotic events in tobacco BY-2 protoplasts. Molecular Plant.

[ref-7] Boothroyd JC, Dubremetz J-F (2008). Kiss and spit: the dual roles of *Toxoplasma* rhoptries. Nature Reviews Microbiology.

[ref-8] Byrt PN, Irving HR, Grant BR (1982). The effect of cations on zoospores of the fungus *Phytophthora cinnamomi*. Journal of General Microbiology.

[ref-9] Carruthers VB, Sibley LD (1997). Sequential protein secretion from three distinct organelles of *Toxoplasma gondii* accompanies invasion of human fibroblasts. European Journal of Cell Biology.

[ref-10] Cerenius L, Andersson MG, Söderhäll K, Lamour K, Kamoun S (2009). *Aphanomyces astaci* and crustaceans. Oomycete genetics and genomics. diversity, interactions, and research tools.

[ref-11] Chappie JS, Mears JA, Fang S, Leonard M, Schmid SL, Milligan RA, Hinshaw JE, Dylewski DP (2011). A pseudoatomic model of the dynamin polymer identifies a hydrolysis-dependent powerstroke. Cell.

[ref-12] Dearnaley JDW, Maleszka J, Hardham AR (1996). Synthesis of zoospore peripheral vesicles during sporulation of *Phytophthora cinnamomi*. Mycological Research.

[ref-13] Essid M, Gopaldass N, Yoshida K, Merrifield C, Soldati T (2012). Rab8a regulates the exocyst-mediated kiss-and-run discharge of the *Dictyostelium* contractile vacuole. Molecular Biology of the Cell.

[ref-14] Fulop T, Radabaugh S, Smith C (2005). Activity-dependent differential transmitter release in mouse adrenal chromaffin cells. Journal of Neuroscience.

[ref-15] Gabor BK, O’Gara ET, Philip BA, Horan DP, Hardham AR (1993). Specificities of monoclonal antibodies to *Phytophthora cinnamomi* in two rapid diagnostic assays. Plant Disease.

[ref-16] Gan PHP, Rafiqi M, Hardham AR, Dodds PN (2010). Effectors of biotrophic fungal plant pathogens. Functional Plant Biology.

[ref-17] Gan PHP, Shan WX, Blackman LM, Hardham AR (2009). Characterization of cyclophilin-encoding genes in *Phytophthora*. Molecular Genetics & Genomics.

[ref-18] Gubler F, Hardham AR (1988). Secretion of adhesive material during encystment of *Phytophthora cinnamomi* zoospores, characterized by immunogold labelling with monoclonal antibodies to components of peripheral vesicles. Journal of Cell Science.

[ref-19] Gubler F, Hardham AR (1990). Protein storage in large peripheral vesicles in *Phytophthora* zoospores and its breakdown after cyst germination. Experimental Mycology.

[ref-20] Harata NC, Aravanis AM, Tsien RW (2006). Kiss-and-run and full collapse fusion as modes of exo-endocytosis in neurosecretion. Journal of Neurochemistry.

[ref-21] Hardham AR (1985). Studies on the cell surface of zoospores and cysts of the fungus *Phytophthora cinnamomi*: the influence of fixation on patterns of lectin binding. Journal of Histochemistry and Cytochemistry.

[ref-22] Hardham AR (1987). Ultrastructure and serial section reconstruction of zoospores of the fungus *Phytophthora cinnamomi*. Experimental Mycology.

[ref-23] Hardham AR, Talbot NJ (2001). Investigations of oomycete cell biology. Molecular and cell biology of filamentous fungi: a practical approach.

[ref-24] Hardham AR, Cahill DM, Cope M, Gabor BK, Gubler F, Hyde GJ (1994). Cell surface antigens of *Phytophthora* spores: biological and taxonomic characterization. Protoplasma.

[ref-25] Hardham AR, Hyde GJ (1997). Asexual sporulation in the Oomycetes. Advances in Botanical Research.

[ref-26] Hardham AR, Suzaki E (1986). Encystment of zoospores of the fungus, *Phytophthora cinnamomi*, is induced by specific lectin and monoclonal antibody binding to the cell surface. Protoplasma.

[ref-27] He L, Wu X-S, Mohan R, Wu L-G (2006). Two modes of fusion pore opening revealed by cell-attached recordings at a synapse. Nature.

[ref-28] Hyde GJ, Lancelle S, Hepler PK, Hardham AR (1991). Freeze substitution reveals a new model for sporangial cleavage in *Phytophthora*, a result with implications for cytokinesis in other eukaryotes. Journal of Cell Science.

[ref-29] Jaiswal JK, Rivera VM, Simon SM (2009). Exocytosis of post-Golgi vesicles is regulated by components of the endocytic machinery. Cell.

[ref-30] Kats LM, Black CG, Proellocks NI, Coppel RL (2006). *Plasmodium* rhoptries: how things went pear-shaped. Trends in Parasitology.

[ref-31] Koeck M, Hardham AR, Dodds PN (2011). The role of effectors of biotrophic and hemibiotrophic fungi in infection. Cellular Microbiology.

[ref-32] Kono M, Hermann M, Loughran NB, Cabrera A, Engelberg K, Lehmann C, Sinha D, Prinz B, Ruch U, Heussler V, Spielmann T, Parkinson J, Gilberger TW (2012). Evolution and architecture of the inner membrane complex in asexual and sexual stages of the malaria parasite. Molecular Biology and Evolution.

[ref-33] Lamour K (2013). Phytophthora. A global perspective.

[ref-34] Lamour K, Kamoun S (2009). Oomycete genetics and genomics. Diversity, interactions, and research tools.

[ref-35] Li D, Zhao Z, Huang Y, Lu Z, Yao M (2013). PsVPS1, a dynamin-related protein, is involved in cyst germination and soybean infection of *Phytophthora sojae*. PLoS ONE.

[ref-36] MacDonald PE, Braun M, Galvanovskis J, Rorsman P (2006). Release of small transmitters through kiss-and-run fusion pores in rat pancreatic β cells. Cell Metabolism.

[ref-37] Macia E, Ehrlich M, Massol R, Boucrot E, Brunner C, Kirchhausen T (2006). Dynasore, a cell-permeable inhibitor of dynamin. Developmental Cell.

[ref-38] McDonald KL, Webb RI (2011). Freeze substitution in 3 hours or less. Journal of Microscopy.

[ref-39] Mendoza L, Lamour K, Kamoun S (2009). *Pythium Insidiosum* and mammalian hosts. Oomycete genetics and genomics. diversity, interactions, and research tools.

[ref-40] Obermüller S, Lindqvist A, Karanauskaite J, Galvanovskis J, Rorsman P, Barg S (2005). Selective nucleotide-release from dense-core granules in insulin-secreting cells. Journal of Cell Science.

[ref-41] Pagni M, Ioannidis V, Cerutti L, Zahn-Zabal M, Jongeneel CV, Hau J, Martin O, Kuznetsov D, Falquet L (2007). MyHits: improvements to an interactive resource for analyzing protein sequences. Nucleic Acids Research.

[ref-42] Rafiqi M, Gan PHP, Ravensdale M, Lawrence GJ, Ellis JG, Jones DA, Hardham AR, Dodds PN (2010). Internalization of flax rust avirulence proteins into flax and tobacco cells can occur in the absence of the pathogen. The Plant Cell.

[ref-43] Robertson EJ, Anderson VL, Phillips AJ, Secombes CJ, Diéguez-Uribeondo J, Van West P, Lamour K, Kamoun S (2009). *Saprolegnia*-fish interactions. Oomycete genetics and genomics. diversity, interactions, and research tools.

[ref-44] Robinson DG, Oliviusson P, Hinz G (2005). Protein sorting to the storage vacuoles of plants: a critical appraisal. Traffic.

[ref-45] Robold AV, Hardham AR (1998). Production of species-specific monoclonal antibodies that react with surface components on zoospores and cysts of *Phytophthora nicotianae*. Canadian Journal of Microbiology.

[ref-46] Robold AV, Hardham AR (2005). During attachment *Phytophthora* spores secrete proteins containing thrombospondin type 1 repeats. Current Genetics.

[ref-47] Sam-Yellowe TY, Shio H, Perkins ME (1988). Secretion of *Plasmodium falciparum* rhoptry protein into the plasma membrane of host erythrocytes. Journal of Cell Biology.

[ref-48] Siddiqui FA, Dhawan S, Singh S, Singh B, Gupta P, Pandey A, Mohmmed A, Gaur D, Chitnis CE (2013). A thrombospondin structural repeat containing rhoptry protein from *Plasmodium falciparum* mediates erythrocyte invasion. Cellular Microbiology.

[ref-49] Sigrist CJA, de Castro E, Cerutti L, Cuche BA, Hulo N, Bridge A, Bougueleret L, Xenarios I (2013). New and continuing developments at PROSITE. Nucleic Acids Research.

[ref-50] Simpson AGB, Inagaki Y, Roger AJ (2006). Comprehensive multigene phylogenies of excavate protists reveal the evolutionary positions of “primitive” eukaryotes. Molecular Biology and Evolution.

[ref-51] Škalamera D, Hardham AR (2006). PnCcp, a *Phytophthora nicotianae* protein containing a single complement control protein module, is sorted into large peripheral vesicles in zoospores. Australasian Plant Pathology.

[ref-52] Suckale J, Solimena M (2010). The insulin secretory granule as a signaling hub. Trends in Endocrinology & Metabolism.

[ref-53] Taraska J, Perrais D, Ohara-Imaizumi M, Nagamatsu S, Almers W (2003). Secretory granules are recaptured largely intact after stimulated exocytosis in cultured endocrine cells. Proceedings of the National Academy of Sciences of the United States of America.

[ref-54] Thiel G, Kreft M, Zorec R (2009). Rhythmic kinetics of single fusion and fission in a plant cell protoplast. Annals of the New York Academy of Sciences.

[ref-55] Topolska AE, Lidgett A, Truman D, Fujioka H, Coppel RL (2004). Characterization of a membrane-associated rhoptry protein of *Plasmodium falciparum*. Journal of Biological Chemistry.

[ref-56] Tropea JE, Cherry S, Nallamsetty S, Bignon C, Waugh DS (2007). A generic method for the production of recombinant proteins in *Escherichia coli* using a dual hexahistidine-maltose-binding protein affinity tag. Methods in molecular bology Vol. 363 macromolecular crystallography protocols Vol. 1 preparation and crystallization of macromolecules.

[ref-57] Tsuboi T, McMahon HT, Rutter GA (2004). Mechanisms of dense core vesicle recapture following “kiss and run” (“cavicapture”) exocytosis in insulin-secreting cells. Journal of Biological Chemistry.

[ref-58] van Kempen GTH, van der Leest HT, van den Berg RJ, Eilers P, Westerink RHS (2011). Three distinct modes of exocytosis revealed by amperometry in neuroendocrine cells. Biophysical Journal.

[ref-59] Vida TA, Emr SD (1995). A new vital stain for visualizing vacuolar membrane dynamics and endocytosis in yeast. Journal of Cell Biology.

[ref-60] Weise R, Kreft M, Zorec R, Homann U, Theil G (2000). Transient and permanent fusion of vesicles in *Zea mays* coleoptile protoplasts measured in the cell-attached configuration. Journal of Membrane Biology.

[ref-61] Zhang Z, Wu Y, Wang Z, Dunning FM, Rehfuss J, Ramanan D, Chapman ER, Jackson M (2011). Release mode of large and small dense-core vesicles specified by different synaptogamin isoforms in PC12 cells. Molecular Biology of the Cell.

[ref-62] Zuccala ES, Gout AM, Dekiwadia C, Marapana DS, Angrisano F, Turnbull L, Riglar DT, Rogers KL, Whitchurch CB, Ralph SA, Speed TP, Baum J (2012). Subcompartmentalisation of proteins in the rhoptries correlates with ordered events of erythrocyte invasion by the blood stage malaria parasite. PLoS ONE.

